# Predictive Attributes for Developing Long COVID—A Study Using Machine Learning and Real-World Data from Primary Care Physicians in Germany

**DOI:** 10.3390/jcm12103511

**Published:** 2023-05-17

**Authors:** Roman Kessler, Jos Philipp, Joanna Wilfer, Karel Kostev

**Affiliations:** 1 Max Planck Institute for Human Cognitive and Brain Sciences, 04103 Leipzig, Germany; 2 IQVIA, 60549 Frankfurt, Germany

**Keywords:** COVID-19, long COVID, machine learning, gradient boosting classifier

## Abstract

(1) In the present study, we used data comprising patient medical histories from a panel of primary care practices in Germany to predict post-COVID-19 conditions in patients after COVID-19 diagnosis and to evaluate the relevant factors associated with these conditions using machine learning methods. (2) Methods: Data retrieved from the IQVIA^TM^ Disease Analyzer database were used. Patients with at least one COVID-19 diagnosis between January 2020 and July 2022 were selected for inclusion in the study. Age, sex, and the complete history of diagnoses and prescription data before COVID-19 infection at the respective primary care practice were extracted for each patient. A gradient boosting classifier (LGBM) was deployed. The prepared design matrix was randomly divided into train (80%) and test data (20%). After optimizing the hyperparameters of the LGBM classifier by maximizing the F2 score, model performance was evaluated using several test metrics. We calculated SHAP values to evaluate the importance of the individual features, but more importantly, to evaluate the direction of influence of each feature in our dataset, i.e., whether it is positively or negatively associated with a diagnosis of long COVID. (3) Results: In both the train and test data sets, the model showed a high recall (sensitivity) of 81% and 72% and a high specificity of 80% and 80%; this was offset, however, by a moderate precision of 8% and 7% and an F2-score of 0.28 and 0.25. The most common predictive features identified using SHAP included COVID-19 variant, physician practice, age, distinct number of diagnoses and therapies, sick days ratio, sex, vaccination rate, somatoform disorders, migraine, back pain, asthma, malaise and fatigue, as well as cough preparations. (4) Conclusions: The present exploratory study describes an initial investigation of the prediction of potential features increasing the risk of developing long COVID after COVID-19 infection by using the patient history from electronic medical records before COVID-19 infection in primary care practices in Germany using machine learning. Notably, we identified several predictive features for the development of long COVID in patient demographics and their medical histories.

## 1. Introduction

COVID-19 is the global pandemic of the 21st century. As of 21 February 2023, there have been approximately 757 million confirmed cases of COVID-19 worldwide, including 6.9 million deaths [1]. In many COVID-19 patients, symptoms persist for at least several months. In a systemic review and meta-analysis of 50 studies, the prevalence of long COVID-19 symptoms 28 days to 12 months after COVID-19 infection was 54% in hospitalized individuals and 34% in non-hospitalized individuals [2]. The proportion of individuals affected by long COVID-19 symptoms has decreased since 2021 due to the emergence of milder COVID-19 variants [3].

An increasing number of decisions in medical applications are being made on the basis of machine learning (ML) algorithms. In view of this, COVID-19 research is also focusing on the development of machine learning algorithms to optimize the prediction of COVID-19 [4] and estimate COVID-19 vaccination side effects [5] or the risk of death as a result of COVID-19 for patients in hospital intensive care units (ICU) [6]. When it comes to long COVID, a study successfully predicted long COVID conditions mainly based on sociodemographic variables and symptom severity during acute COVID-19 infection using a case–control design [7]. In addition, one study was conducted to identify potential long COVID patients using gradient boosting models that had been trained on patients treated in a specialized long COVID clinic [8] and applied to patient cohort data from a US COVID-19 database. Nevertheless, the authors themselves state that their study does not represent all population strata, especially because it does not include people who are not insured and people who are unable to afford medical treatment in the US. The disadvantages associated with their data also apply to electronic medical records (EMR) such as in the database used in the present study. For example, these records only document patient visits to general practitioners (GPs) and do not document patient visits to different specialty practices or hospitals. Additionally, the data are skewed towards patients who visit their general practitioners regularly. Nevertheless, the advantage of having defined trajectories for a cross-section of the population allows the model to include chronic and acute diseases, sick leave days, treatments, and other information. Finally, GPs are the primary point of contact for patients suffering from long COVID.

In the present exploratory study, we used data comprising patient medical histories from a panel of primary care practices in Germany to predict long COVID symptoms in patients after COVID-19 diagnosis and to evaluate the relevant factors associated with these symptoms using ML methods. To our knowledge, this is one of the first studies using electronic medical records to identify potential features predictive for the development of long COVID.

## 2. Materials and Methods

### 2.1. Data Set

The data used in the present study were retrieved from the IQVIA^TM^ Disease Analyzer database, which contains information from approximately 3% of primary care practices in Germany, and includes demographics, diagnoses, and prescription data, in an anonymized format, retrieved from the computer systems of cooperating practices. Previous research has shown that the panel is representative of primary care practices in Germany [9].

### 2.2. Study Population

Patients with at least one COVID-19 diagnosis (ICD-10: U07.1 or U08.9) between January 2020 and July 2022 were selected for inclusion in this study. Of these patients, a subpopulation was then formed comprising patients with one recorded certain diagnosis of long COVID (ICD-10: U09.9). Data on age, sex, and the complete history of diagnoses and prescription data at the respective primary care practice were extracted for each patient before their first COVID-19 infection. A categorical variable representing each primary care practice ID was also added.

We applied several filters when selecting patients for analysis. First, the distances between all patients’ first COVID-19 diagnoses and the long COVID diagnoses were calculated. The 75% quartile of the distribution (86 days) was considered the minimum distance to the last available timepoint in the database. All patients who received their first COVID-19 diagnosis less than 86 days prior to the last available timepoint of the database were therefore excluded from further analysis. In addition, patients with less than 30 days between their first recorded COVID-19 diagnosis and the long COVID diagnosis were excluded from further analysis. Patient history was analyzed prior to the first COVID-19 diagnosis to exclude COVID-19-related diagnoses or medication as predictors. Furthermore, patients with a documented long COVID diagnosis but no prior documented COVID-19 infection were excluded from the dataset, as the date of the first COVID-19 infection is necessary to determine the cutoff for the patient’s history.

Finally, 272,588 patients were available for the ML models, 5440 of whom had a long COVID diagnosis.

### 2.3. Feature Preparation

Data cleansing and preprocessing were conducted using SAS (version 9.4, SAS Institute, Cary, NC, USA). Each diagnosis was classified into third-level ICD-10 categories based on the classification of the Federal Institute for Drugs and Medical Devices [10]. Similarly, prescriptions were classified into third-level ATC categories based on the anatomical chemical classification (ATC) of the European Pharmaceutical Market Research Association (EphMRA) [11]. After this, the number of diagnoses and prescriptions within the respective ICD-10 or ATC category across the entire patient history were counted to assess patients’ general utilization of the health care system. To reduce the number of features for training, the 50 most frequent ICD-10 and ATC categories were selected within the present patient population.

We added the number of COVID-19 diagnoses per patient as another feature. Distinct diagnoses were assumed if the time between two diagnoses was more than four weeks. While for the other features we only looked at the patient’s history prior to the first COVID-19 infection, for this feature we looked at additional COVID-19 diagnoses after the first COVID-19 diagnosis but before a potential long COVID diagnosis.

Further features were again based on the history available for each patient. We included the time span between the first and the last record of a patient (visibility days). Patients with visibility of under 100 days were excluded. The median visibility among the remaining patients was 5.9 years (10% quantile: 1.3 years, 90% quantile: 17.5 years). Explicitly including visibility as a feature allows the classifier to account for different lengths of patient histories in its decision. In addition, the number of sick leave days was calculated based on the medical history. Similarly, the number of recorded hospital referrals was calculated. These newly created variables were normalized by relating them to the length of the respective patient visibility.

Using data from the Robert Koch Institute (RKI), the corresponding relative probabilities of each virus strain (wild type, Alpha, Beta, Delta, various Omicron subtypes) were assigned to the first COVID-19 diagnosis of a patient [12]. The current vaccination rates of the population were assigned in a similar fashion based on the date of the first COVID-19 infection of a patient to estimate the probability of vaccination-related immunity [13]. Two vaccination rates were used representing the basic immunization rate (two shots administered) and the first booster (third shot administered). This modeling using external data was necessary because only a small portion of COVID-19 vaccinations is reported in our initial data, as the vaccination campaign in Germany was distributed across fixed and mobile vaccination centers and vaccinations were not administered solely by GPs.

The data were entered into a design matrix, with each row representing one patient and each column representing one variable as described above. The target variable was a binary vector considering a long COVID diagnosis (=1) or no long COVID diagnosis (=0) after COVID-19 diagnosis. All further processing was conducted in Python (v. 3.9.15) using sklearn (v. 1.1.3). Categorical variables were one-hot encoded. Where values were missing in the categorical variables, the redundant column representing the missing value was dropped from the data set (i.e., sex, <0.1%). In the case of count variables, missing values were imputed with zeros. The prepared design matrix was randomly divided into two data sets: the train data (80%) and the test data (20%). Missing values in the age variable (<0.1%) were imputed with the median age derived from the train data.

### 2.4. Training

In this study, we deployed the light gradient boosting machine (LGBM), a performant gradient boosting algorithm based on decision trees [14]. It was used because algorithms of this kind are widely used to identify potential features and disease outcomes [8], and are supposed to perform better than, e.g., neural networks in tabular data [15]. In addition, the LGBM algorithm used here is a well-established classifier which is used in a variety of classification approaches [5,16,17]. It is equally performant to other boosting classifiers and, therefore, is a good choice for the classification of long COVID in primary care practices [18].

An LGBM binary classifier was trained using the Python lightGBM (v. 3.3.3 [14]) package. Several hyperparameters were optimized using a grid search with 5-fold cross-validation within the train data set (Table S1). Hyperparameters were optimized to maximize the F2 score of the model. The F2 score is a weighted harmonic mean of precision and recall, whereby recall is weighted double relative to precision [19]. A higher weighting of recall was applied in order to acknowledge potential false-negative labels in the train data so as to correct for patient hopping and diagnoses at other practices in particular.

Model performance was evaluated on the test data set illustrating a contingency matrix, precision, recall, specificity, F2 score, ROC-AUC, and accuracy metrics.

### 2.5. Feature Importance

Shapley Additive Explanation (SHAP) values were calculated (v. 0.41.0 [20]) to evaluate the contribution of the individual features to the model’s performance. SHAP is a generic game theoretic approach allowing for the interpretation of features for any machine learning model [20]. Contrary to many other approaches, SHAP allows the direction of the effects of features onto the target variable to be interpreted. SHAP takes into consideration the contribution of each feature in conjunction with all possible combinations of other features in the model, and therefore returns an integrated view of feature importance.

The one-hot encoded variable describing the practice identifiers comprised many columns in the design matrix, as several thousand practices were included. Therefore, the SHAP values were summarized across practices within each row (patient) of the design matrix to estimate the overall effect of the category “practice,” rather than the contribution of each single practice [21].

## 3. Results

### 3.1. Model Performance

**Figure 1:**
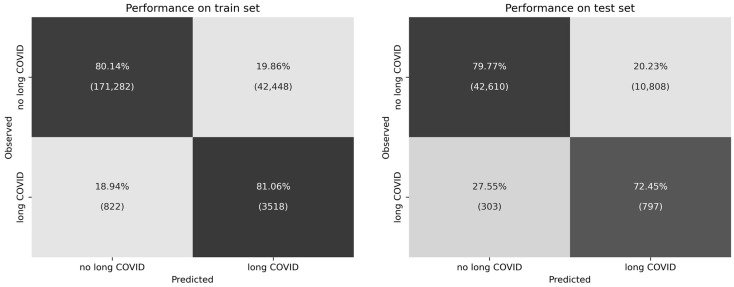
Contingency matrices of model performance on the train and test data sets. Left: train data set. Right: test data set. The *y*-axes represent the “true” observed data labels, i.e., no long COVID diagnosis, or long COVID diagnosis. The *x*-axes represent the data labels predicted by the model. True negatives (correctly identified patients without long COVID diagnoses) are illustrated in the upper left. True positives (correctly identified long COVID patients) are illustrated in the lower right. Each cell contains percentages relating to the total proportion of patients with or without long COVID diagnoses labeled in the data (i.e., row-wise). Brackets contain the total amount of patients in each cell.

Across the entire train data set, the model showed an accuracy of 80%, a precision of 8%, a recall (sensitivity) of 81%, a specificity of 80%, an ROC-AUC of 0.9, and an F2 score of 0.28. On the test data set, the model showed an accuracy of 80%, a precision of 7%, a recall of 72%, a specificity of 80%, an ROC-AUC of 0.84, and an F2 score of 0.25. Note that the data set classes were imbalanced. Accuracy and ROC-AUC are therefore not particularly suitable as criteria for model effectiveness, but are reported nevertheless for the convenience of the reader. The contingency matrices for train and test data sets are illustrated in Figure 1. A total of 81% and 72% of long COVID patients were identified correctly by the model from the train and test data sets, while 80% and 80% of patients, respectively, without a long COVID diagnosis were identified correctly by the model in the train and test data sets. All further inferences will be made based on the test data set only.

### 3.2. Feature Importance

**Figure 2:**
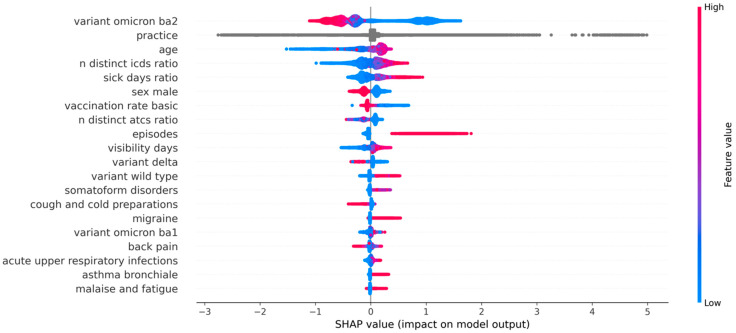
Feature importance as estimated via SHAP. Only the 20 most important features are displayed in descending order (top to bottom). SHAP values are illustrated on the *x*-axis. Higher feature values (red) represent data points with higher variable expression. Lower feature values (blue) represent data points with lower variable expression. Gray values represent the influence of the categorical practice IDs. When higher feature values (red) are distributed to the positive range of the *x*-axis, and lower feature values (blue) are distributed to the negative range of the *x*-axis, the variable expression is positively associated with the development of long COVID. By contrast, if higher feature values distribute to the negative range and lower feature values distribute to the positive range, the feature is negatively associated with the development of long COVID.

SHAP was used to estimate feature importance. Figure 2 illustrates the 20 most important features and the relative impact of a variable expression for the development of long COVID in our data. Feature values are displayed in either red or blue. When higher feature values (red) are associated with positive SHAP values (positive range on the *x*-axis), and lower feature values (blue) are associated with negative SHAP values (negative range on the *x*-axis), the variable expression is positively associated with the development of long COVID. By contrast, if higher feature values distribute to the negative range and lower feature values distribute to the positive range, the feature is negatively associated with the development of long COVID.

**Figure 3:**
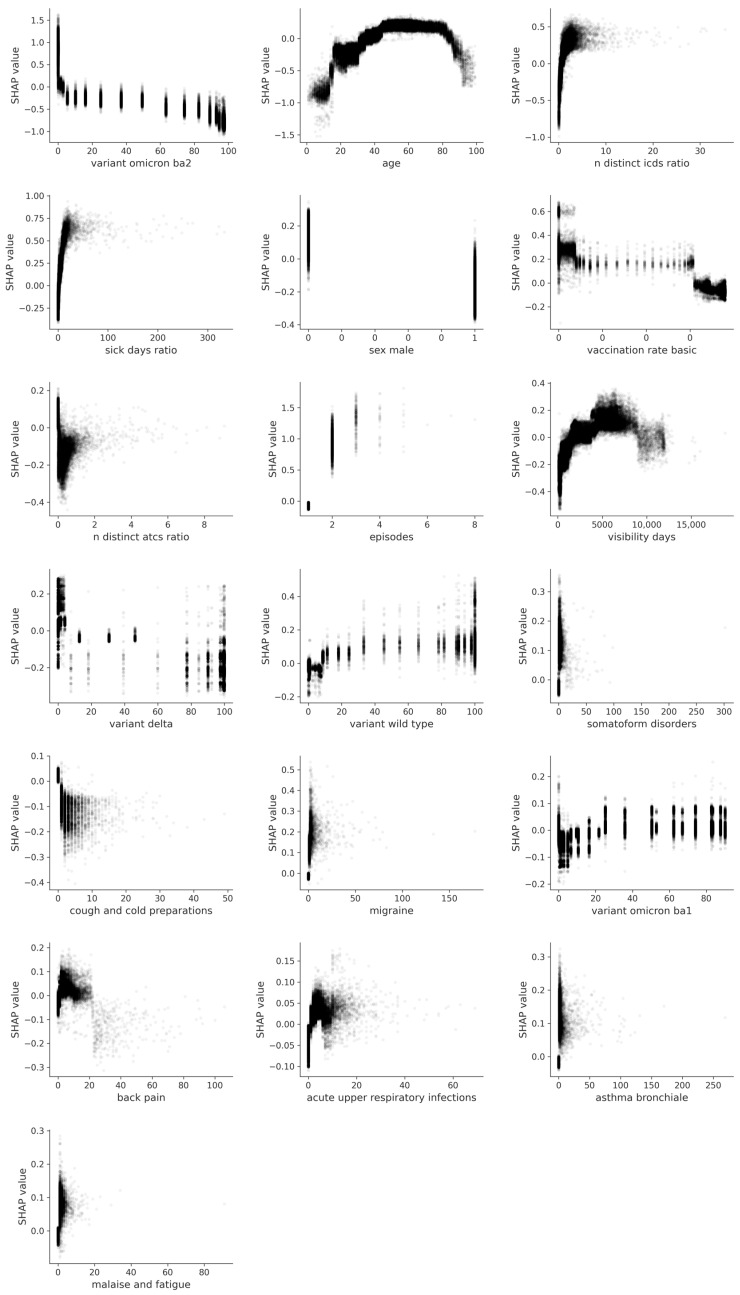
Dependency of long COVID on feature expression. The 19 most important features (when the categorical variable “practice” is excluded) according to the SHAP analysis (Figure 2) are displayed from top left to bottom right. For each feature, the corresponding SHAP value (*y*-axis) is related to the respective variable expression (*x*-axis).

For the top 19 features (there are actually 20 features, but we are excluding the summarized categorical feature “practice”) identified in our SHAP analysis, we also illustrated the SHAP values as a function of the variable expression (Figure 3).

#### 3.2.1. SARS-CoV-2 Variants

The most important feature in our analysis was variant Omicron-BA2, indicating that patients with a COVID infection at a time with a higher proportion of variant Omicron-BA2 had a lower probability of developing long COVID (Figure 2 and Figure 3). Conversely, this highlights that patients with COVID infection at a time when the proportion of variant Omicron-BA2 was lower (and, in turn, the probability of other variants was higher) were more likely to develop long COVID. While the influence of the Delta variant is similar (Figure 2 and Figure 3), it is less strong, as highlighted by its lower feature importance (order on the *y*-axis in Figure 2). Furthermore, the relative proportion of the wild type variant of SARS-CoV-2 showed a positive association with long COVID, with higher probability of being infected with the wild type strain pointing towards an increased risk of developing long COVID (Figure 2 and Figure 3).

This is more clearly reflected in Figure 3, which depicts the dependence of SHAP-values on feature expression. Here, the SHAP values are shown as a function of the variable expression, i.e., the proportion of the respective strain in all sequenced samples for a given point in time. The SHAP value for the wild type variant was higher when the proportion for the wild type in the population was highest, indicating a higher probability of long COVID when the probability of being infected with the wild type strain (on first COVID diagnosis) was higher. However, the opposite effect can be observed for the Omicron-BA2 variant. The highest SHAP values are found where the proportions of the variant were lowest (Figure 2 and Figure 3). A mixture of the two is shown for the Delta variant, with a tendency to show lower SHAP values at higher proportions of the variant (Figure 2 and Figure 3). For the Omicron-BA1 variant, the effect is rather similar to that of the wild type variant, with higher proportions of Omicron-BA1 at the time of infection associated with higher probability of long COVID. Note that the stepwise representation of the proportions of the variants in Figure 3 results from the weekly data used from the RKI tables. In these tables, the proportions of the strains can change rapidly between successive weeks.

#### 3.2.2. Sociodemographic and Practice Effects, and General Diagnosis and Medication Counts

To also control for the effect of the individual physician on long COVID diagnosis, the sum of SHAP values of all practice IDs was consolidated, resulting in practice being the second most important feature (Figure 3). The third most important feature was patient age. Age had a strong impact on the model prediction, with low age values leading to negative SHAP values, whereas high age values led to higher SHAP values and, therefore, a higher probability of long COVID. When looking at the feature expressions, the SHAP values were distributed as an inverted U (Figure 3). Higher SHAP values—indicative of a higher probability of long COVID—were associated with an age of between 30 and 80 years. Higher and lower age showed negative SHAP values, with very low values before an age of 15 and after an age of 80.

The ratio of distinct ICD-10 classes and the sick day ratio, as the fourth and fifth most important features, show a similar distribution of SHAP values in Figure 2. For both, the SHAP value increased with higher feature expressions, indicating a positive association between the development of long COVID and having multiple different diagnoses before COVID-19 infection, as well as having more sick days before COVID-19 infection (both relative to the observation period of a particular patient). Furthermore, SHAP values for the number of COVID episodes showed a high positive impact on the model. The number of episodes is a proxy for the number of COVID infections (cf. Section 2). For this purpose, distinct diagnoses were counted no earlier than 4 weeks after the previous COVID diagnosis. This includes patients with either a long-lasting infection or recurring infections. The analysis of the dependence plots in Figure 3 demonstrates that as few as two episodes already lead to higher SHAP values and, therefore, a higher probability of long COVID, with each additional episode increasing the risk. Longer visibility of a patient within our database also contributed to higher SHAP values.

Male sex reduced the probability of developing long COVID, as this feature showed an inverted pattern of SHAP values around the *x*-axis (Figure 2). This is also illustrated in Figure 3, as the “1” depicts male sex and is therefore associated with a lower SHAP value than patients with female or unknown (“0”) sex. The risk of long COVID was also reduced if patients had most likely received the basic vaccination (Figure 2), which is defined as the first two vaccination shots. This can also be seen in the dependence plots (Figure 3), where the increasing rate of full vaccination across Germany is related to lower SHAP values. In addition, the distinct number of different ATC classes in patient history was slightly negatively associated with a higher risk of developing long COVID (Figure 2). The dependence analysis here did not provide a clear picture (Figure 3). Higher SHAP values were slightly associated with a lower number of distinct ATC classes. However, with a very low number of distinct ATC classes (i.e., 0), both low and high SHAP values can be discerned.

#### 3.2.3. ICD-10 Classes

Within the 20 most important features, features describing ICD-10 classes were ranked lowest (Figure 2). The feature expression of each ICD-10 class stands for the number of the respective diagnosis in the patient history before the first recorded COVID-19 infection. Within the ICD-10 classes, somatoform disorders (ICD-10: F45) were the most important feature (Figure 2). The SHAP values suggest that patients diagnosed with somatoform disorders had a higher risk of developing long COVID (Figure 2). SHAP values for back pain (ICD-10: M54) showed high feature values on the positive part of the *x*-axis and on the negative part of the *x*-axis, making the interpretation less clear in Figure 2 and Figure 3. For migraine (ICD-10: G43), asthma (ICD-10: J45), and malaise and fatigue (ICD-10: R53), positive feature values also tended towards positive SHAP values, indicating a higher probability of long COVID (Figure 2). In the dependence analysis (Figure 3), back pain and acute upper respiratory infections (ICD-10: J06) had similar SHAP value distributions, with lower SHAP values connected to a low number of diagnosis codes. SHAP values increased generally with an increasing number of the respective diagnosis codes in the respective patient history. Somatoform disorders, malaise and fatigue, asthma, and migraine all had a broad variety of SHAP values associated with already low numbers of diagnosis codes, accumulating around 0.

**Figure 4:**
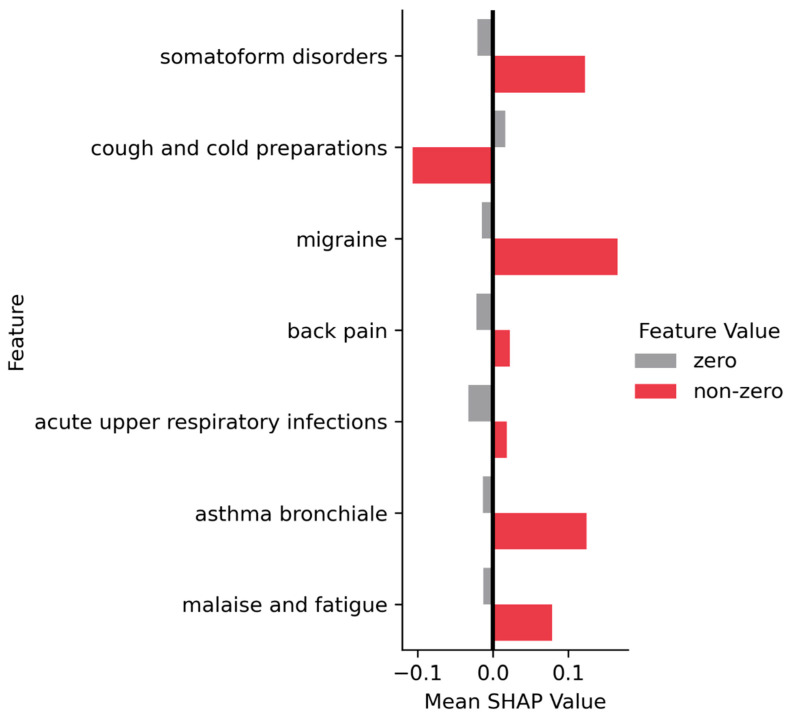
Average SHAP values for diagnosis and medication codes, averaged within two respective patient groups with one or more occurrences of the code (*non-zero*) or with no occurrence (*zero*). Top to bottom order reflects feature importance. *X*-axis illustrates mean SHAP value for the respective subgroup. Note that the absolute length of the bars does not directly indicate the importance of the feature.

To better determine the effects of finding particular diagnoses (and medications) in a patient history on the probability of developing long COVID, we also dichotomized each ICD and ATC code into patients with either a zero or non-zero amount of a particular diagnosis code or medication code in their histories. We then averaged the SHAP values of all patients in each group (zero and non-zero, respectively). Figure 4 illustrates the mean SHAP values for each of the most predictive diagnoses and medication codes, averaged for patients with and without the code, respectively. Figure 4 clearly illustrates that the effects point mainly in the positive direction, i.e., where a patient history includes a particular diagnosis, the SHAP values tend to be positive, while otherwise, they tend to be negative. This circumvents the limitations of the dependence plots (Figure 3), where it is difficult to infer the exact density of the SHAP values in particular regions. For most ICD-10 codes in Figure 3, there is a point mass of data points visually hidden with a negative SHAP value at a feature expression of zero.

#### 3.2.4. ATC Classes

Only one ATC class was predictive enough to be included as one of the 20 most important features for the model. The ATC class R05C (cough-related products including antihistamines and bronchodilators) shows a negative impact on the model. Patients receiving products in this ATC class are less likely to develop long COVID (Figure 2, Figure 3 and Figure 4). The higher number of prescriptions of this class is associated with decreasing SHAP values, and therefore a lower probability of long COVID.

## 4. Discussion

In this retrospective, exploratory study including more than 270,000 patients with COVID-19, a good prediction of long COVID was achieved using an LGBM classifier. This is, to the best of our knowledge, the first investigation on the prediction of potential features increasing the risk for developing Long-COVID after COVID-19 infection in primary care practices in Germany. Additionally, particularly novel is the use of electronic medical record data for the prediction of long COVID, having been performed only a few times, such as in [8]. Further, this is the first study that focuses on the first point of medical contact of patients.

The first finding of our study is the good performance of the LGBM classifier. In the train dataset, 81% of long COVID patients and 80% of non-long COVID patients were correctly identified by the model. In the test dataset, the proportions were 72% and 80%, respectively. Aktar et al. also successfully attempted to predict clinical outcomes in COVID-19 patients based on different peripheral blood values, using several ML models to identify blood parameters that can predict the risk of serious illness among COVID-19 patients [22]. Furthermore, Sudre et al. predicted long COVID conditions based on symptoms during the first week of illness and sociodemographic factors [7] using a matched case–control design and achieved good model performance. Because the data set we used is unbalanced with respect to diagnoses (long COVID vs. no long COVID), and because it does not comprise case–control matched groups, direct comparison of model performance to many other studies is difficult. However, there was no marked drop in model goodness between train and test data, suggesting good generalizability of our model.

The second finding of our study is the identification of a number of important features. Patients who were diagnosed with COVID-19 at a time with a higher proportion of the Omicron-BA2 variant had a lower risk of developing long COVID, whereas a higher proportion of the wild type variant of SARS-CoV-2 was positively associated with the risk of developing long COVID. This finding is partly in line with other published research. Du et al. performed a systematic review and meta-analysis including a total of 51 studies with 33,573 patients to evaluate the characteristics of long COVID caused by different SARS-CoV-2 variants. While authors suggested that there was no significant difference between different variants in terms of long COVID incidence, symptoms of long COVID differed strongly depending on SARS-CoV-2 variant. For example, ≥1 general symptoms and fatigue occurred most commonly in patients infected with the Alpha variant, followed by patients with the wild type strain, and less often among patients with the Omicron variant [23].

The second most important feature was the practice in which a patient was treated. The high importance of this variable is interesting, but not surprising, as it captures different diagnostic styles in medical practices. Especially with such new diagnostic codes and for such a heterogeneous clinical picture as long COVID, for which guidelines and information change rapidly, individual doctors can come to very different assessments as to whether or not a patient suffers from long COVID. Furthermore, some of the practices might also (begin to) treat long COVID with a focus, while other practices might not approach a diagnosis.

In our study, age 30–80 and female sex were associated with a higher risk of long COVID. Interestingly, in another study based on the same database but using logistic regression to analyze associations between different variables and long COVID, age 45–60 was associated with a 2.1 times higher risk of long COVID compared with age group 18–30; female sex was associated with a 1.2 times higher risk of developing long COVID. However, further variables such as asthma and somatoform disorders were also positively associated with long COVID [24]. Although the association between sex and long COVID is still insufficiently understood, several other studies also reported that the prevalence of long COVID was higher in women than in men [7,25,26,27,28,29].

Somatoform disorders which were associated with a higher risk of long COVID in our study can be characterized by symptoms such as back pain, headache, fatigue, dizziness, and shortness of breath without an adequate medical explanation. COVID-19 patients who have a coexisting somatoform disorder may harbor a belief that these symptoms are due to long COVID [30]. Another study from Poland which assessed factors associated with prolonged symptoms in non-hospitalized patients with COVID-19 demonstrated that female sex, asthma, history of myocardial infarction, and severity of symptoms in the acute phase of COVID-19 were the predictors of long COVID [31]. Two of these predictors (female sex, asthma) were found among the top 20 features in our study. Another study also used symptom severity in an early phase of COVID infection to successfully predict long COVID in a case–control designed analysis [7]. In addition to symptom severity and symptom quantity, female sex, age, and asthma were also predictive for long COVID in their study, nicely converging on our findings.

In our study, we observed a positive association between multimorbidity (multiple different diagnoses or a higher number of sick days before COVID-19 infection) with subsequent long COVID. Wilk et al. analyzed data from different European countries and found that multimorbid individuals had an increased risk of experiencing symptoms of long COVID, identifying a slightly increased relative risk of 1.12 for such individuals [32]. However, multimorbidity is known to impact COVID-19 severity and mortality as well as the risk of long COVID [27,33]. On the other hand, polypharmacy was negatively associated with the risk of long COVID in our study.

A further finding of our study is that a higher likelihood of COVID-19 vaccination was negatively associated with the risk of long COVID. Although we used a proxy for vaccination (cf. Methods), this finding is not surprising and was already reported in a systematic review by Notarte et al. [34]. Based on case–control and cohort studies included in this review, the authors suggested that vaccination before SARS-CoV-2 infection could reduce the risk of subsequent long COVID [34]. This finding is quite prevalent, such as, for example, in a recent meta-analysis [29].

Further variables included in the top 20 features identified by our model such as migraine, malaise and fatigue, or back pain include symptoms which can also occur as symptoms of long COVID (headache, back pain, fatigue). These symptoms may worsen after COVID-19 infection and, thus, transition to long COVID. In general, many physical and also mental disorders have been shown to increase risk for long COVID [27,28].

Prescription of cough medication and polypharmacy are two further variables that are included in our top 20 features. Considering the effect of polypharmacy, one could speculate, for example, that it counteracts the negative effect of multimorbidity when therapies have been claimed. However, making a statement is difficult, and an investigation of the time course and composition of the therapies would be necessary to understand the effect. The only single drug class that appears to be predictive and simultaneously protective was cough medication. Patients receiving cough medications might be more prone to the hazard of bronchial diseases. Therefore, those patients might have additional medications for the treatment of bronchial diseases mitigating acute COVID-19 symptoms, which are in turn predictive for long COVID [7]. Further research is needed here as well. All other individual drug groups did not make it onto the list of highly predictive features, unlike the individual diagnostic codes, some of which were represented.

The strengths of this study are the inclusion of more than 270,000 patients, the use of data from clinical practice, and the use of ML methodology. However, the study is also subject to several limitations, which should be acknowledged at this point. First, all diagnoses relied on ICD-10 codes pre-COVID-19 infection only, and no data were available on symptoms of long COVID. Second, long COVID may sometimes have been diagnosed in specialized practices (e.g., pneumology) or hospitals, and some of the related data may have gone undocumented in the Disease Analyzer database, potentially leading to an underestimation of the prevalence of this condition. The prevalence of long COVID observed in our study was much lower than that in published investigations, probably due to the rare use of the ICD-10 codes U09.9 in the first year after the beginning of the pandemic and also due to our exclusion criteria. Third, no medications used for COVID-19 therapy were analyzed, as these are usually given in hospitals and are only administered for severe courses of COVID-19. Fourth, viral variants were not determined individually for patients, but rather assigned based on the predominant variant at the time the patient was first diagnosed with COVID-19. Since we did not have any information about the genome sequence of the virus, the estimation via the time of infection and the inclusion of the epidemiological situation was the most obvious way to estimate the strain. However, an interpretation of the virus strains should be approached with caution and confirmed with actual sequencing studies. Fifth, we trained the model to achieve a high recall, and have compromised on a lower level of precision, therefore allowing for a high proportion of false positives to achieve a very low proportion of false negatives. The focus could have been set differently to achieve a better accuracy of the model. Since the main reason for us was to identify predictive features, we deliberately set a high recognition rate of long COVID patients in our model to correct for the underrepresentation of this diagnosis at the GP. Finally, limitations come with the analysis of real-world data, which are temporally unstructured and full of missing values compared with studies designed in a matched case–control fashion. Insights, however, highly converged on other studies using matched study designs and different data sources.

## 5. Conclusions

The present study describes an initial investigation of the prediction of potential features increasing the risk of developing long COVID after COVID-19 infection in primary care practices in Germany using machine learning on the patient history before COVID-19 infection retrieved from electronic medical record data. Importantly, we identified several predictive features for the development of long COVID in patient demographics and their medical histories.

## Data Availability

The data that support the findings of this study are available from the corresponding author on reasonable request.

## Supplementary Materials

The following supporting information can be downloaded at: https://www.mdpi.com/article/10.3390/jcm12103511/s1, Table S1: Hyperparameters were optimized in grid search. Asterisks indicate the hyperparameters of the optimal model, which were used for further analysis. Learning rate was set to 0.05. The remaining hyperparameters were set to their default values.
