# Supplementary material for: Predictive Attributes for Developing Long COVID—A Study Using Machine Learning and Real-World Data from Primary Care Physicians in Germany
Source: J Clin Med. 2023 May 17;12(10):3511. doi: 10.3390/jcm12103511 (PMC10219004; doi:10.3390/jcm12103511)
Supplement: Supplementary file 1 [file jcm-12-03511-s001.zip › jcm-2314596-supplementary.pdf]

Table S1. Hyperparameters were optimized in grid search. Asterisks indicate the hyperparameters of the optimal model, which were used for further analysis. Learning rate was set to 0.05. The remaining hyperparameters were set to their default values.

.

Hyperparameter	Values
num_leaves	7, 31*
reg_alpha	0.1*, 0.5
reg_lambda	0.1*, 0.5
min_data_in_leaf	5*, 30, 100
n_estimators	50, 100*
